# Hsa-miR-4277 Decelerates the Metabolism or Clearance of Sorafenib in HCC Cells and Enhances the Sensitivity of HCC Cells to Sorafenib by Targeting *cyp3a4*

**DOI:** 10.3389/fonc.2021.735447

**Published:** 2021-07-26

**Authors:** Xi He, Huiwei Sun, Qiyu Jiang, Yantao Chai, Xiaojuan Li, Zhijie Wang, Bing Zhu, Shaoli You, Boan Li, Junfeng Hao, Shaojie Xin

**Affiliations:** ^1^ Chinese People’s Liberation Army (PLA) Medical School, Beijing, China; ^2^ Department of Liver Disease of Chinese PLA General Hospital, The Fifth Medical Center of Chinese PLA General Hospital, Beijing, China; ^3^ Institute of Infectious Disease, Department of Infectious Disease, The Fifth Medical Center of Chinese PLA General Hospital, Beijing, China; ^4^ Department of Clinical Laboratory, The Fifth Medical Center of Chinese PLA General Hospital, Beijing, China; ^5^ Department of Nephrology, Jin Qiu Hospital of Liaoning Province/Geriatric Hospital of Liaoning Province, Shenyang, China

**Keywords:** advanced hepatocellular carcinoma, cytochrome P450 3A4, miR-4277, chemoresistance, sorafenib, metabolism or clearance

## Abstract

Increasing evidence has shown that the metabolism and clearance of molecular targeted agents, such as sorafenib, plays an important role in mediating the resistance of HCC cells to these agents. Metabolism of sorafenib is performed by oxidative metabolism, which is initially mediated by CYP3A4. Thus, targeting CYP3A4 is a promising approach to enhance the sensitivity of HCC cells to chemotherapeutic agents. In the present work, we examined the association between CYP3A4 and the prognosis of HCC patients receiving sorafenib. Using the online tool miRDB, we predicted that has-microRNA-4277 (miR-4277), an online miRNA targets the 3’UTR of the transcript of *cyp3a4*. Furthermore, overexpression of miR-4277 in HCC cells repressed the expression of CYP3A4 and reduced the elimination of sorafenib in HCC cells. Moreover, miR-4277 enhanced the sensitivity of HCC cells to sorafenib *in vitro* and *in vivo*. Therefore, our results not only expand our understanding of CYP3A4 regulation in HCC, but also provide evidence for the use of miR-4277 as a potential therapeutic in advanced HCC.

## Introduction

Though many advances have been made in the treatment of advanced HCC, it remains a major challenge for China’s public health (1–3). In China, more than 80 million people suffer from viral liver disease or related acute and chronic liver diseases (1). These patients have a high risk of developing HCC (1, 4). Furthermore, most HCC patients are diagnosed with advanced disease, so they are not candidates for radical treatment strategies like surgery or liver transplantation (5–7). At present, drug treatment strategies for advanced HCC are limited, they are mainly based on various molecular targeted therapeutics (8–10), or immune checkpoint inhibitors targeting PD-1/PD-L1 (programmed cell death-1/programmed cell death ligand-1) (11–13). Among them, molecular-targeted agents like sorafenib have been used in clinical treatment for many years, though several problems remain: the efficacy of sorafenib varies based on the patient, and resistance is common; sorafenib treatment often induces serious side effects (14, 15); newer agents such as lenvatinib, regorafenib, and carbozantinib (8–10), have the same chemical parent ring structure (1-(4- (pyridin-4-yloxy)phenyl) urea) as sorafenib. Therefore, it is important to understand the mechanisms of resistance to sorafenib and other agents, and to study and explore sensitization strategies for HCC cells to molecular targeted agents. This will not only provide more choice for the patient, but it will also help improve the efficacy of combination therapies using targeted agents and/or immune checkpoint inhibitors.

The metabolism and clearance mechanisms of sorafenib and other exogenous agents in HCC cells are crucial factors in the development of HCC chemoresistance (16, 17). *Via* a systemic analysis, Feng et al. (18) and Shao et al. (19) found that sorafenib can act as a ligand/agonist to activate PXR/NR1I2 (pregnane X receptor/nuclear receptor subfamily 1 group I member 2) and induce expression of downstream genes involved in chemoresistance, including *cyp3a4* and *abcb1* (ATP-binding cassette, sub-family B, member 1). Ultimately, this accelerates elimination of the therapeutic and results in drug resistance through negative feedback regulation (18, 19). Although related studies have expanded our understanding of PXR in HCC, much remains unclear; PXR is not the only metabolism-related nuclear receptor in HCC cells, and the CAR/NR1I3 (constitutive androstane receptor/nuclear receptor subfamily 1 group I member 3) may have similar functions to PXR (20, 21). By inhibiting the activity of PXR alone, CAR may have a compensatory effect on the function of PXR. Moreover, targeting oxidative metabolism, which is mediated by CYP3A4 in the initial step of sorafenib elimination in HCC cells, represents a promising approach to enhance the sensitivity of these cells to targeted agents (22, 23). This makes CYP3A4 more advantageous to target compared with PXR or CAR. Both PXR and CAR mediate the expression of CYP3A4 to eliminate sorafenib. By inhibiting CYP3A4, compensatory effects between PXR and CAR can be avoided.

MicroRNA is a type of small non-coding RNA transcribed by RNA polymerase II (24–27). In mammalian cells, miRNA can directly affect the 3’UTR of the target mRNA to degrade it in a sequence-specific manner and silence gene expression (28–30). Because of this feature, miRNAs are widely used as anti-cancer therapeutics. By predicting miRNAs that target certain sets of oncogenes, one can identify novel anti-cancer miRNAs that can be added to lentiviral particles to reduce expression of the target oncogene and sensitize cells to targeted agents. Our study used the online tool miRDB to identify miR-4277, a potential repressor of CYP3A4 expression. We infected HCC cells with lentiviral particles containing pre-miR-4277 and confirmed the effect of miR-4277 on CYP3A4 and the elimination of sorafenib.

## Materials and Methods

### Cell Lines and Reagents

The HCC cell lines, MHCC97-H, HepG2, BEL7402 or SMMC7721, were grown in our lab and described previously (18, 19). The clinical specimens of advanced HCC were also descripted in our previous work (18, 19). The use of human subjects was approved by the ethics committee of the Fifth Medical Center, General Hospital of Chinese PLA (People’s Liberation Army). All assays were carried out in accordance with the Helsinki Declaration. Sorafenib, lenvatinib, cabozantinib, regorafenib, anlotinib, and apatinib were chemically synthesized by Dr. Shuang Cao at the Wuhan Institute of Technology, Wuhan City, Hubei Province of China. The potential cyp3a4’s inhibitor, ketoconazole, amprenavir or diltiazem, was also gifts from Dr. Shuang Cao at the Wuhan Institute of Technology, Wuhan City, Hubei Province of China. All agents were initially prepared as powders purified to >99% by using the HPLC (high performance liquid chromatography) (**Supplemental Table 1**). The miR-4277 was a microRNA potentially targeting to *cyp3a4*’s 3’UTR *via* an online tool, miRDB, and the full-length sequences of has-pre-miR-4277, wild-type *cyp3a4* and *cyp3a4*, and miR-4277 mutated at its targeting sites (two targeting sites of miR-4277 located in the 3’UTR of *cyp3a4*: 1^st^ site of 496^th^ – 503th nt [a 8^mer^ site]; 2^nd^ site of 984^th^ – 991^st^ nt [a 8^mer^ site]) for the 3’UTR were chemically synthesized and prepared as lentiviral particles: (1) Luc-1 (the luciferase reporter with the wild type of the 1^st^ miR-4277 targeting site), (2) Luc-2 (the luciferase reporter with the wild type of the 2^nd^ miR-4277 targeting site), (3) Luc-3 (the luciferase reporter with the wild type of the 1^st^ and 2^nd^ miR-4277 targeting site), (4) Luc-4 (the luciferase reporter with the wild type of the 2^nd^ and the mutated 1^st^ miR-4277 targeting site), (5) CYP3A4 mutations (CYP3A4^Mut1^ [the vector of *cyp3a4* with the mutation of the 1^st^ miR-4277 targeting site], CYP3A4^Mut2^ [the vector of *cyp3a4* with the mutation of the 2^nd^ miR-4277 targeting site], or CYP^Mut^ [the vector of *cyp3a4* with the mutation of the 1^st^ and 2^nd^ miR-4277 targeting site]).

### Quantitative PCR

The endogenous mRNA levels of *cyp3a4* in HCC clinical specimens were identified using quantitative polymerase chain reaction (qPCR) in accordance with methods described by Wang et al. (20) and Ma et al. (20, 25). The primers used were: (1) *cyp3a4*, forward sequence 5’-CCGAGTGGATTTCCTTCAGCTG-3’, reverse sequence 5’-TGCTCGTG GTTTCATAGCCAGC-3’; (2) *β-actin*, forward sequence 5’-CACCATTGGCAATGA GCGGTTC-3’, reverse sequence 5-AGGTCTTTGCGGAT GTCCACGT-3’; (3) PXR/NR1I2 (31), forward sequence 5’-CCCACCTCAGA AGACAAAGC-3’, reverse sequence, 5’-GAACCCCAGACCCTACACAA-3’ (4); CAR/NR1I3 (31): forward sequence, 5’-TACTGTGCTTCGTGCTCCTG-3’, Reverse sequence, 5’-CCTGG TCTTCGGGTTCAAG-3’. The results (the relative expression level [folds of β-Actin]) of PXR or CAR was shown as heat-map.

### Western Blot

MHCC97-H cells were cultured and TACT transfected with plasmids, such as miR-4277 or *cyp3a4*; the cells were then harvested and proteins were extracted as previously described by Wei et al. (32) and Jia et al. (32, 33). Antibodies against CYP3A4 (Cat. No.: ab124921) and β-Actin (Cat. No.: ab8226) were purchased from the Abcam Corporation, Cambridge, UK). The images of western blot was quantitatively analyzed by Image J Software (National Institutes of Health [NIH], Bethesda, Maryland, USA).

### Assessment of Sorafenib Elimination in HCC Cells

HCC cells were used to examine the rate of elimination of sorafenib (18, 19, 34, 35). For cell-based experiments, HCC cells were treated with 1 μmol/L of sorafenib for 12 h. After treatment, the cells were harvested at a series of time-points. For *in vivo* experimentation, HCC cells were cultured and subcutaneously injected into mice to generate tumors. When tumor volume reached 2000 mm^3^, a solution of sorafenib was directly injected into the tumors. After injection, the tumors were excised at a series of time-points. Next, sorafenib was extracted from MHCC97-H cells or tumors using the acetonitrile (ACN). The sustaining amount of sorafenib at each time-point was measured using liquid chromatography–mass spectrometry/mass spectrometry (LC-MS/MS) and the *in vitro*/*in vivo* half-life of sorafenib was determined (18, 19, 34, 35).

### Assessment of Cell Survival

After transfection or treated with potential inhibitor of *cyp3a4*, HCC cells were treated with a series concentrations of sorafenib, lenvatinib, cabozantinib, regorafenib, anlotinib, or apatinib (10 μmol/L, 3 μmol/L, 1 μmol/L, 0.3 μmol/L, 0.1 μmol/L, 0.03 μmol/L, or 0.01 μmol/L). After 48 h, MTT was performed to measure ell survival, and the IC_50_ value of each agent was calculated (36–38).

### Nude Mouse Tumor Model

All animal experiments were reviewed approved by the Institutional Animal Care and Use Committee, the Fifth Medical Center, Chinese PLA. All animal experiments were performed in accordance with the UK Animals (Scientific Procedures) Act, 1986 and the associated guidelines. Female nude mice were purchased from the Si-Bei-Fu Corporation, Beijing China. Following transfection, MHCC97-H cells were prepared as a single-cell suspension and subcutaneously injected into nude mice (39, 40). The mice orally received sorafenib once every two days. Tumors from the mice were harvested, and the volumes and weights were measured (39, 40).

### Statistical Analysis

SPSS 9.0 statistical software (IBM Corporation, Armonk, NY, USA) was used for all statistical analyses. Statistics were calculated using two-way ANOVA with the Bonferroni correction. IC_50_ values and the half-lie values (t_1/2_ values) were calculated using Origin software (Origin 6.1; OriginLab Corporation, Northampton, MA, USA). The *P < 0.05 being statistically significant between groups. The heat-map of the qPCR results were obtained according to the methods by Zhou et al. (41) and Yin et al. (42).

## Results

### High Levels of *cyp3a4* mRNA Expression Are Associated With Poor Prognosis in HCC Patients Receiving Sorafenib

**Figure 1:**
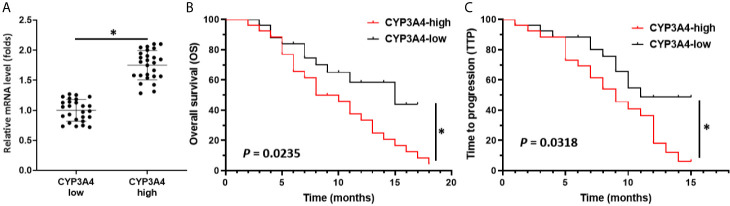
High levels of *cyp3a4* is associated with poor prognosis in advanced HCC. **(A)** The endogenous mRNA levels of *cyp3a4* were assessed in 52 clinical specimens from patients with advanced HCC. According to the median expression level of *cyp3a4* in HCC tissue samples, patients were divided into two groups: a *cyp3a4* high expression group [*cyp3a4*-high] and a *cyp3a4* low expression group [*cyp3a4*-low]) **(B**, **C)**. Survival analysis included analysis of TTP and OS. *P < 0.05.

Though it has been suggested that *cyp3a4* participates in the resistance of HCC cells to sorafenib, the clinical significance of *cyp3a4* requires further analysis. As shown in **Figure 1**, the endogenous mRNA levels of *cyp3a4* were examined in 52 clinical specimens from patients with advanced HCC (**Figure 1** and **Table 1**). According to the median expression level of *cyp3a4* in HCC tissue samples, patients were divided into two groups: a *cyp3a4* high expression group [*cyp3a4*-high] and a *cyp3a4* low expression group [*cyp3a4*-low]) (**Figure 1A**). We then performed survival analysis (**Figures 1B, C**). The prognosis of patients in the *cyp3a4* high expression group [*cyp3a4*-high] treated with sorafenib, as measured by TTP (time to progress) and OS (overall survival), was significantly worse than that of the *cyp3a4* low expression group [*cyp3a4*-low]) (**Figures 1B, C** and **Table 1**). Therefore, high levels of *cyp3a4* appear to be associated with poor prognosis of HCC patients receiving sorafenib.

**Table 1:** CYP3A4 expression and clinical outcome of sorafenib treatment.

	CYP3A4 mRNA expression		P
	High (n = 26)	Low (n = 26)	
TTP	9.0	11.0	0.032
	6.1-11.8 (M)	9.5-12.9 (M)	
OS	8.0	15.0	0.023
	4.8-11.2 (M)	6.9-23.1 (M)	

TTP, time to progress; OS, overall survival; PR, partial remission; CR, complete remission; SD, stable of disease; M, months.

### miR-4277 Represses the Expression of *cyp3a4* by Targeting its 3’UTR

**Figure 2:**
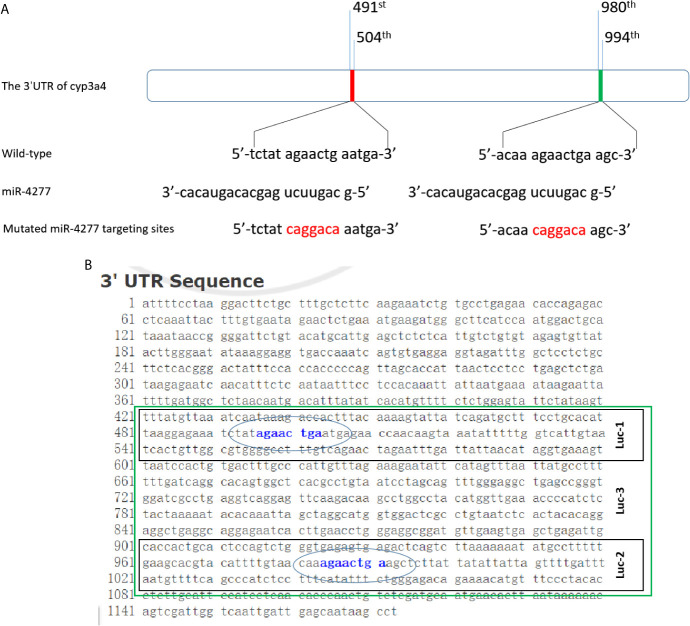
miR-4277 is predicted to target the 3’UTR of *cyp3a4*. **(A)** The sequences of miR-4277 and *cyp3a4* are shown as schematic diagrams. **(B)** The luciferase reporters containing the 3’UTR region of cyp3a4 containing the miR-4277 binding site are shown as a schematic diagram.

**Figure 3:**
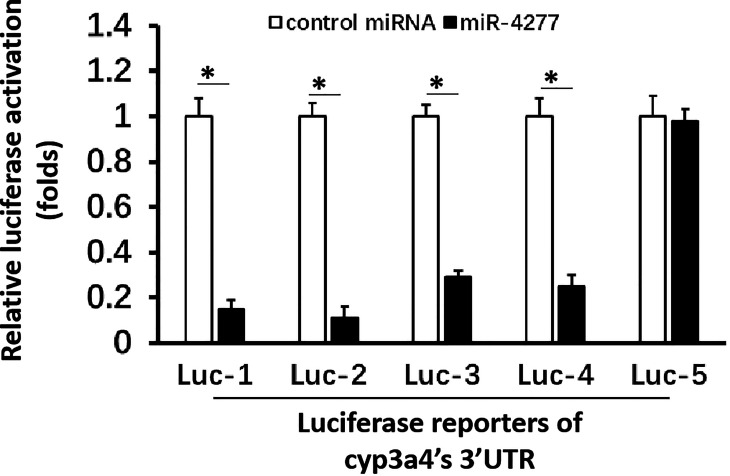
miR-4277 represses the activation of *cyp3a4*’s 3’UTR luciferase reporters in MHCC97-H cells. The effect of miR-4277 on the activation of Luc-1 (the luciferase reporter with the wild type of the 1^st^ miR-4277 targeting site) or Luc-2 (the luciferase reporter with the wild type of the 2^nd^ miR-4277 targeting site), Luc-3 (the luciferase reporter with the wild type of the 1^st^ and 2^nd^ miR-4277 targeting site), Luc-4 (the luciferase reporter with the wild type of the 2^nd^ and the mutated 1^st^ miR-4277 targeting site), or Luc-5 (the luciferase reporter with the wild type of the 2^nd^ and the mutated 1^st^ miR-4277 targeting site) was examined. The results are shown as histograms. *P < 0.05.

To explore CYP3A4 as a potential anti-cancer target, miR-4277 was identified *via* the online tool miRDB as a microRNA that could potentially target *cyp3a4*. The potential binding sites of miR-4277 in the 3′-UTR of *cyp3a4* as well as in wild type *cyp3a4* and *cyp3a4* with mutated miR-4277 binding sites are shown in **Figure 2A**. To confirm the effects of miR-4277 on *cyp3a4* expression, we used a luciferase reporter construct for *cyp3a4* and transfected cells with miR-4277. As shown in **Figures 2B** and **3**, overexpression of miR-4277 repressed the activation of Luc-1 (the luciferase reporter with the wild type of the 1^st^ miR-4277 targeting site), Luc-2 (the luciferase reporter with the wild type of the 2^nd^ miR-4277 targeting site), Luc-3 (the luciferase reporter with the wild type of the 1^st^ and 2^nd^ miR-4277 targeting site), and Luc-4 (the luciferase reporter with the wild type of the 2^nd^ and the mutated 1^st^ miR-4277 targeting site). Moreover, miR-4277 did not affect the activation of Luc-5 (the luciferase reporter with the wild type of the 2^nd^ and the mutated 1^st^ miR-4277 targeting site) (**Figure 3**).

**Figure 4:**
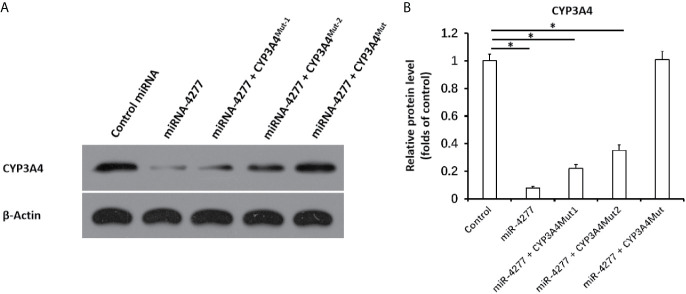
miR-4277 represses the expression of CYP3A4 in MHCC97-H cells. The effect of miR-4277 on wild type CYP3A4 or CYP3A4 mutations (CYP3A4Mut1 [the vector of *cyp3a4* with the mutation of the 1^st^ miR-4277 targeting site], CYP3A4^Mut2^ [the vector of *cyp3a4* with the mutation of the 2^nd^ miR-4277 targeting site], or CYP^Mut^ [the vector of *cyp3a4* with the mutation of the 1^st^ and 2^nd^ miR-4277 targeting site]) in MHCC97-H cells was measured by western blot. The results are shown as blots **(A)** or the quantitative results **(B)**. *P < 0.05.

Next, the effect of miR-4277 on the CYP3A4 protein expression was examined *via* western blot. As shown in **Figure 4**, miR-4277 not only inhibited the protein expression of the endogenous CYP3A4 in MHCC97-H cells, but also the expression of CYP3A4^MUT1^ or CYP3A4^Mut2^. Transfection of miR-4277 did not affect the expression of CYP3A4^MUT^ (**Figure 4**). Therefore, these results suggest that miR-4277 targets the 3’UTR of *cyp3a4* in a sequence specific manner.

### miR-4277 Reduces the Elimination of Sorafenib in HCC Cells

Our results above suggest that miR-4277 represses the expression of CYP3A4 in MHCC97-H cells; therefore, we assessed whether miR-4277 could affect the elimination of sorafenib *via* LC-MS/MS. As shown in **Table 2**, overexpression of miR-4277 reduces elimination of sorafenib in cultured MHCC97-H cells; the half-life (t_1/2_) of sorafenib in MHCC97-H cells was also decreased in the presence of miR-4277 (**Table 2**). Transfection of CYP3A4^Mut^ but not CYP3A4^Mut1^ or CYP^Mut2^ blocked the effect of miR-4277 on the reduction in t_1/2_ of sorafenib (**Table 2**). Therefore, miR-4277 blocked elimination of sorafenib in HCC cells by targeting the 3’UTR of *cyp3a4*.

**Table 2:** The effect of miR-4277 on Sorafenib in cultured MHCC97-H cells.

Groups	*t_1/2_* of Sorafenib (hours)	*IC_50_* of Sorafenib (μmol/L)
control miRNA	21.60 ± 1.34	0.70 ± 0.09
miR-4277	38.46 ± 9.97	0.15 ± 0.01
miR-4277 + CYP3A4^Mut-1^	22.11 ± 6.59	0.22 ± 0.14
miR-4277 + CYP3A4^Mut-2^	24.68 ± 8.63	0.35 ± 0.05
miR-4277 + CYP3A4^Mut^	19.88 ± 2.71	0.92 ± 0.20

### miR-4277 Enhances the Sensitivity of HCC Cells to Targeted Agents

To further examine the effect of miR-4277 on *cyp3a4* in HCC cells, the effects of other targeted agents were assessed by MTT. As shown in **Table 2**, overexpression of miR-4277 enhanced the sensitivity of MHCC97-H cells to sorafenib, with a decrease in its IC_50_ value. Transfection of CYP3A4^Mut^ but not CYP3A4^Mut1^ or CYP^Mut2^ blocked the effect of miR-4277 on the reduction in t_1/2_ value for sorafenib. Similar results were obtained for five other targeted agents, including regorafenib, lenvatinib, anlotinib, cabozantinib, and apatinib (**Table 3**). Therefore, miR-4277 appears to enhance the sensitivity of HCC cells to multiple targeted agents by targeting the 3’UTR of *cyp3a4*.

**Table 3:** miR-4277 enhanced the sensitivity of MHCC97-H cells to molecular targeted agents, regorafenib, lenvatinib, anlotinib, cabozantinib, or apatinib.

Groups	Regorafenib	Lenvatinib	Anlotinib	Cabozantinib	Apatinib
	*IC_50_* values of the agents on MHCC97-H cells (μmol/L)				
**control miRNA**	0.67 ± 0.49	0.55 ± 0.20	0.75 ± 0.03	0.51 ± 0.19	0.96 ± 0.33
**miR-4277**	0.22 ± 0.04	0.12 ± 0.06	0.10 ± 0.01	0.10 ± 0.04	0.26 ± 0.07
**miR-4277 + CYP3A4^Mut-1^**	0.33 ± 0.32	0.41 ± 0.01	0.30 ± 0.12	0.25 ± 0.06	0.52 ± 0.27
**miR-4277 + CYP3A4^Mut-2^**	0.35 ± 0.09	0.28 ± 0.11	0.45 ± 0.35	0.30 ± 0.21	0.46 ± 0.30
**miR-4277 + CYP3A4^Mut^**	0.71 ± 0.28	0.60 ± 0.20	0.78 ± 0.55	0.56 ± 0.15	0.98 ± 0.37

### miR-4277 Inhibits the Growth and Induces Sensitization of HCC Tumors to Sorafenib in Nude Mice

**Figure 5:**
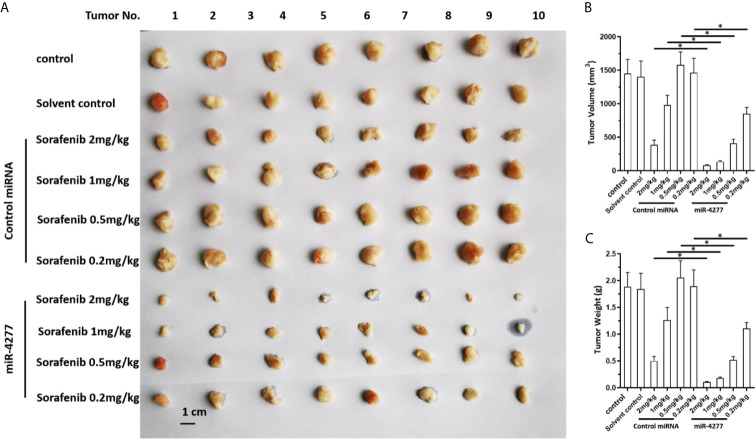
miR-4277 enhances the antitumor effects of sorafenib in inhibiting the growth of HCC cells in a nude mouse model. MHCC97-H cells were transfected with vectors (control miR or miR-4277) and subcutaneously injected into nude mice. The mice then received the indicated dose of sorafenib *via* oral administration. The results are shown as tumor images **(A)**, tumor volumes **(B)**, or tumor weights **(C)**. *P < 0.05.

The effect of miR-4277 to induce sensitivity to sorafenib was further confirmed in a nude mice model. As shown in **Figure 5**, sorafenib inhibited the subcutaneous growth of HCC cells in a dose-dependent manner, and transfected with miR-4277 further enhanced this effect (**Figure 5**).

**Figure 6:**
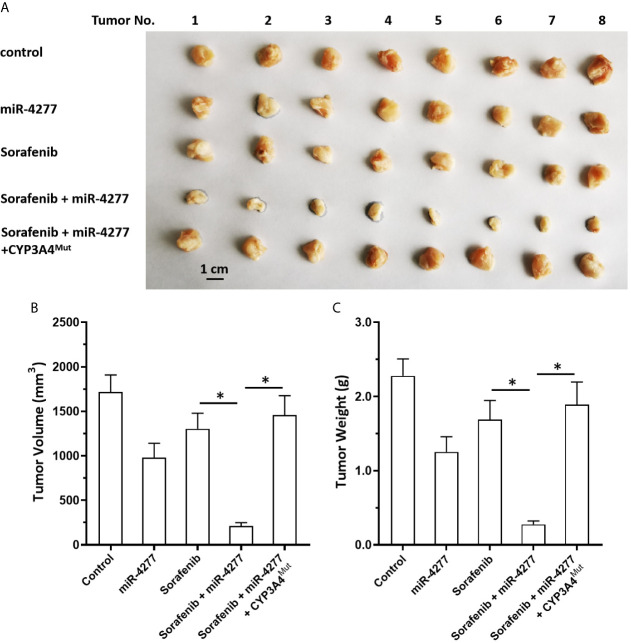
miR-4277 enhances the antitumor effect of sorafenib in a nude mouse model. MHCC97-H cells were transfected with vectors (control miR, miR-4277, or miR-4277 + CYP3A4^Mut^) and subcutaneously injected into nude mice. The mice then received the indicated dose of sorafenib *via* oral administration. The results are shown as tumor images **(A)**, tumor volumes **(B)**, or tumor weights **(C)**. *P < 0.05.

Moreover, the specificity of miR-4277 for *cyp3a4* was examined in our subcutaneous tumor model. As shown in **Figure 6**, MHCC97-H cells were transfected with control, miR-4277, or miR-4277 + CYP3A4^Mut^ (the expression vectors of *cyp3a4* with the mutated binding sites of miR-4277). Transfection of miR-4277 inhibited the subcutaneous growth of MHCC97-H cells, and a 0.5 mg/kg dose of sorafenib had no effect (**Figure 6**). Transfection of miR-4277 enhanced the sensitivity of MHCC97-H cells to sorafenib, whereas CYP3A4^Mut^ blocked the effect of miR-4277 (**Figure 6**).

The elimination of sorafenib in subcutaneous tumors were also examined to further confirm the effect of miR-4277. As shown in **Table 4**, overexpression of miR-4277 reduced the rates of sorafenib elimination in tumor tissues formed by MHCC97-H cells, and the t_1/2_ of sorafenib in these tissues was also decreased. Transfection of CYP3A4^Mut^ but not CYP3A4^Mut1^ or CYP^Mut2^ blocked this effect. These results further confirm the effect of miR-4277 on sorafenib elimination.

**Table 4:** Effect of miR-4277 on the clearance of sorafenib in the subcutaneous tumors formed by MHCC97-H cells.

Groups	*t_1/2_* of Sorafenib (hours)
control miRNA	45.68 ± 5.31
miR-4277	72.79 ± 7.52
miR-4277 + CYP3A4^Mut-1^	52.38 ± 16.19
miR-4277 + CYP3A4^Mut-2^	48.74 ± 28.50
miR-4277 + CYP3A4^Mut^	36.67 ± 12.58

### The Endogenous mRNA Level of PXR and CAR in Clinical Specimens

**Figure 7:**
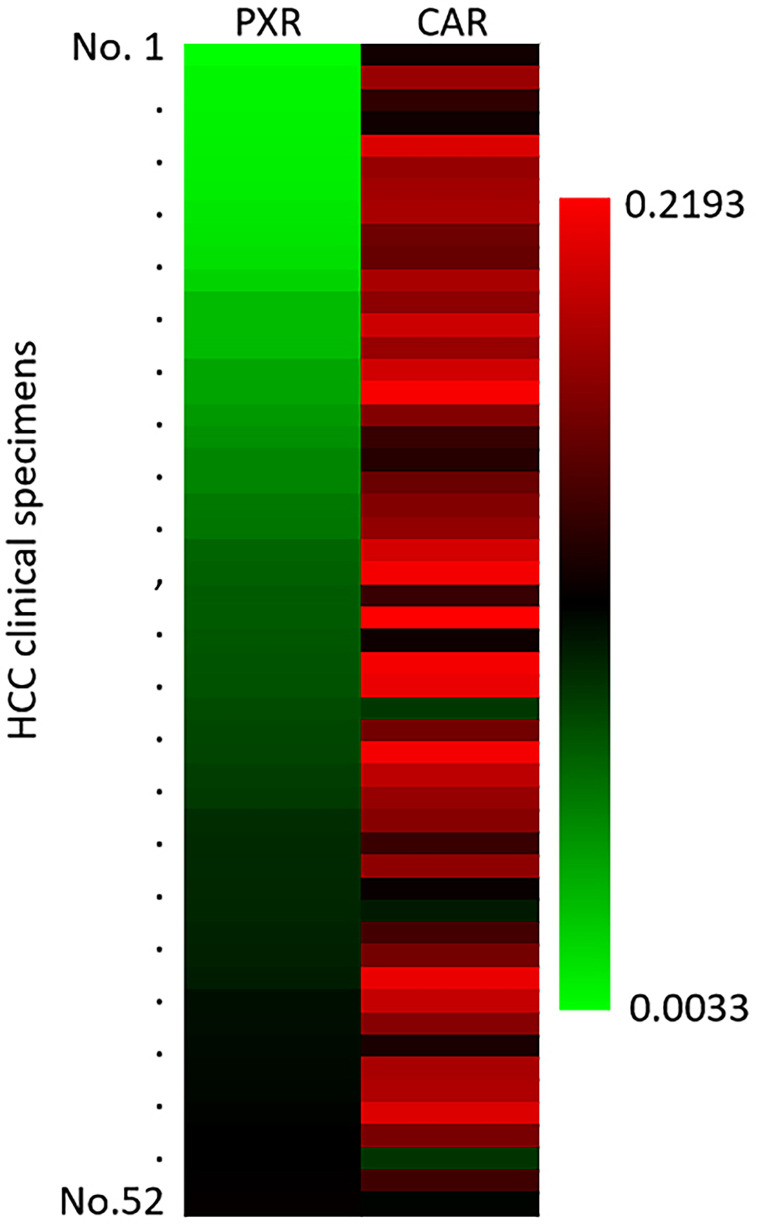
The endogenous expression of PXR or CAR in HCC clinical specimens. The endogenous expression of PXR or CAR in HCC clinical specimens was examined by qPCR. The results were shown as heat-map from the relative expression level (folds of β-Actin).

The above results mainly focus on the roles of miR-4277/*cyp3a4* in HCC cells. To further confirm the clinical significance of *cyp3a4*, the expression level of PXR and CAR in HCC clinical specimens was examined by qPCR. As shown in **Figure 7**, the expression of PXR or CAR were detected in the HCC clinical specimens. The expression level of CAR was also higher than that of PXR (**Figure 7**). Therefore, the endogenous mRNA level of PXR and CAR are positive in HCC clinical specimens and the compensatory effect between PXR and CAR cannot be ignored.

### miR-4277 Also Enhances the Sensitivity of Sorafenib in Some Other HCC Cell Lines

Next, the effect of miR-4277 on sorafenib was examined in some other HCC cell lines. As shown in **Table 5**, transfection with miR-4277 decelerated the metabolism or clearance of sorafenib in HepG2, BEL-7402 or SMMC-7721 cells, and the t_1/2_ values of sorafenib increased, respectively (**Table 5**). Moreover, transfection of miR-4277 also enhances the sensitivity of these three HCC cell lines to sorafenib (**Table 5**), and the IC_50_ values of sorafenib decreased, respectively (**Table 5**). Transfection of CYP3A4^Mut^ almost blocked the effect of miR-4277 on sorafenib’s metabolism/clearance rates or the antitumor activation on HCC cells. Therefore, miR-4277 also enhances the sensitivity of sorafenib in some other HCC cell lines by targeting *cyp3a4*’s 3’UTR.

**Table 5:** The effect of miR-4277 on sorafenib in HCC cells.

Sorafenib on HCC cells	Groups	HepG2	BEL-7402	SMMC-7721
t_1/2_ values (h)	control	30.30 ± 3.31	28.20 ± 8.67	25.70 ± 2.43
	miR-4277	56.43 ± 1.90	42.40 ± 3.33	46.25 ± 2.88
	miR-4277 + CYP3A4^Mut^	25.74 ± 4.47	26.36 ± 3.79	27.39 ± 6.93
IC_50_ values (μmol/L)	control	2.78 ± 0.59	1.79 ± 0.18	1.95 ± 0.54
	miR-4277	0.41 ± 0.07	0.28 ± 0.05	0.73 ± 0.33
	miR-4277 + CYP3A4^Mut^	3.56 ± 0.82	2.88 ± 0.35	1.92 ± 0.55

### The Potential Inhibitor of CYP3A4 Enhances the Sensitivity of MHCC97-H Cells to Sorafenib

Furthermore, the effect of potential inhibitors of cyp3a4 were used. As shown in **Table 6**, treatment of 1μmol/L concentration of ketoconazole, amprenavir or diltiazem, decelerated the metabolism or clearance of sorafenib in MHCC87-H cells, and the t_1/2_ values of sorafenib increased, respectively (**Table 6**). Treatment of these agents also enhances the sensitivity of MHCC97-H cells to sorafenib (**Table 6**), and the IC_50_ values of sorafenib decreased, respectively (**Table 6**). The effect of ketoconazole is much greater than amprenavir or diltiazem. Therefore, the inhibited of CYP3A4 both *via* miR-4277 or inhibitors could enhance the sensitivity of HCC cells to sorafenib.

**Table 6:** The effect of potential inhibitors of CYP3A4 on sorafenib in MHCC97-H cells.

Cell lines	Sorafenib on MHCC97-H	
	t_1/2_ values (h)	IC_50_ values (μmol/L)
Solvent control	23.63 ± 0.70	0.68 ± 0.11
ketoconazole	44.17 ± 2.86	0.08 ± 0.01
amprenavir	33.85 ± 3.92	0.42 ± 0.05
diltiazem	37.40 ± 0.43	0.34 ± 0.10

## Discussion

Currently, targeted therapy remains a first-line choice for patients with advanced HCC; however, the overall clinical benefit of these therapies are unsatisfactory (43–45). It is important to block chemoresistance pathways in HCC to improve sensitivity to targeted agents. Zhu et al. (14) systematically summarized the possible mechanisms of resistance of HCC cells to sorafenib and discerned the compensatory effects of various signal pathways, including the epithelial-mesenchymal transition and signals from the tumor stem cell environment, may increase resistance to sorafenib (14). Although studies like these are beneficial to expand our understanding of sorafenib resistance in advanced HCC, several issues persist. The liver is the body’s regulatory center for exogenous metabolism and clearance (46, 47). HCC arises from hepatocytes, and the metabolism and clearance of exogenous agents may be specific to the ability of HCC cells to tolerate sorafenib (18, 19). Feng et al. (18) showed that sorafenib can function as a ligand/agonist to induce PXR transcription factor activity and accelerate the metabolism and clearance rate of sorafenib. It does this by inducing the expression of drug resistance genes downstream of PXR, such as *cyp3a4* or *abcb1* (ATP-binding cassette, sub-family B, member 1), and through a similar negative feedback mechanism that induces resistance of HCC cells to sorafenib itself. This means that HCC patients with high background expression levels of PXR may not be sensitive to sorafenib (18). With long-term treatment, sorafenib can also induce the activity of PXR in HCC cells and the expression of drug-resistant genes, which ultimately leads to multidrug resistance (18). CYP3A4 is an important regulator of sorafenib metabolism and clearance in HCC cells as it can mediate the oxidative metabolism of sorafenib (48). Our study identified that a microRNA that can act on the 3’UTR of *cyp3a4*: miR-4277. We found that it could down-regulate the expression levels of CYP3A4 in HCC cells, reduce elimination of sorafenib, and ultimately enhance the sensitivity of HCC cells to sorafenib. Therefore, miR-4277 and *cyp3a4* represent ideal targets that can be modulated to overcome the resistance of HCC cells to targeted therapy. Our results also showed that the potential inhibitors of CYP3A4 had the similar effect of miR-4277 on sorafenib in HCC cells. The effect of ketoconazole is significantly stronger than amprenavir or diltiazem (49–55). Since ketoconazole is also considered to be an inhibitor of PXR, this needs to be discussed in depth.

The transcription of *cyp3a4* is mainly mediated by PXR, but it can also regulated by CAR (56–58). In cancerous cells, particularly in HCC, the activity of PXR may also be compensated by CAR. Using miRNA or PXR antagonists alone to down-regulate the activity of PXR may be an inefficient method of fully blocking the resistance of malignant tumor cells to anti-tumor agents. CAR can also induce the resistance of tumor cells to anti-tumor agents. Wang et al. (20) previously used miR-4271 to down-regulate the expression levels of CAR, which significantly enhanced the sensitivity of tumor cells to anlotinib (20). Our results also examined the expression of PXR and CAR in HCC’s clinical specimens. Therefore, we selected CYP3A4 in our study to avoid the compensation effects of PXR and CAR in HCC cells.

miRNA is an important type of non-coding RNA that induces the post-transcriptional silencing of target genes’ expression in a sequence-specific manner by targeting the 3’UTR (26). This feature makes miRNA an ideal strategy for anti-tumor gene therapy; tumors can be treated by cloning the full pre-miRNA sequence into a vector and delivering it in lentiviral particles (26). Transfection of lentiviral particles into tumor cells or *via* intratumor tissue injection can down-regulate the expression levels of certain oncogenes (26). Li et al. (59), Yang et al. (60), and Li et al. (61) showed that miR-140-3p, miR-30c, and miR-148a can inhibit the expression of PXR and CYP3A4, respectively, by acting on the 3’UTR of PXR and reduce the elimination of anti-cancer agents in tumor cells. Our study used an online tool to identify miR-4277 as a regulator of *cyp3a4* expression. This miRNA had the highest score among all miRNAs tested from the miRDB database. We then constructed mutants to confirm the effect of miR-4277 on *cyp3a4*. Transfecting HCC cells with miR-4277 decreased the expression levels of CYP3A4 and enhanced the sensitivity of HCC cells to targeted agents. Besides miR-4277, there are also some other miRNAs could target to *cyp3a4*. Ekström et al. (62), Tang et al. (63), Gill et al. (64), Huang et al. (65), Li et al. (66) and Zastrozhin et al. (67) suggested that the miR-27 family, miR-142, miR-200a, miR-150 or miR-328 could targets to *cyp3a4* (62–67). Therefore, our results extended the knowledge of miRNAs on *cyp3a4*.

It is worth mentioning that in our study, elimination of sorafenib was measured *via* LC-MS/MS. We observed that miR-4277 could prolong the half-life of sorafenib in HCC cells and tissues and reduce its elimination. Several other targeted agents were tested for their ability to kill HCC cells; however, we failed to detect the presence of these agents in HCC cells, mainly due to the lack of efficient protocols for assessing the levels of the compounds. In the future, LC-MS/MS methodology for these and other targeted agents will likely be established well enough to determine the effect of miR-4277 on their elimination rate in HCC cells.

Furthermore, Drug interactions mediated by CYP3A4 are not only closely related to clinical treatment and drug contraindications, but also an important mechanism of anti-tumor drug resistance (68, 69). CYP3A4 is not only closely related to the metabolism and drug resistance of cytotoxic chemotherapy agents such as paclitaxel and camptothecin, but also closely related to the metabolism and drug resistance of molecularly targeted agents such as Imatinib, Gefitinib and Pazopanib (70–75). Therefore, the results of this study can be extended to other types of malignant tumor molecular targeted therapy in the future.

## Data Availability Statement

The original contributions presented in the study are included in the article/**Supplementary Material**. Further inquiries can be directed to the corresponding authors.

## Ethics Statement

The studies involving human participants were reviewed and approved by the ethics committee of the Fifth Medical Center, General Hospital of Chinese PLA. The patients/participants provided their written informed consent to participate in this study. The animal study was reviewed and approved by the Institutional Animal Care and Use Committee, the Fifth Medical Center, Chinese PLA.

## Author Contributions

SX, JH, and BL conceived the main ideas and wrote the paper. XL, BZ, and ZW supervised the study. XH, HS, QJ, YC, and SY developed major methodologies, databases, reagents, and primary experiments. XH, HS, and QJ analyzed different aspects of the results. All authors contributed to the article and approved the submitted version.

## Funding

This work is supported by National Science and Technology Major Project of China, Chinese Government, Chinese Government (new techniques and new schemes for clinical treatment of severe hepatitis B [liver failure]; NO 2017ZX10203201004).

## Conflict of Interest

The authors declare that the research was conducted in the absence of any commercial or financial relationships that could be construed as a potential conflict of interest.

## Publisher’s Note

All claims expressed in this article are solely those of the authors and do not necessarily represent those of their affiliated organizations, or those of the publisher, the editors and the reviewers. Any product that may be evaluated in this article, or claim that may be made by its manufacturer, is not guaranteed or endorsed by the publisher.
