# Supplementary material for: Hsa-miR-4277 Decelerates the Metabolism or Clearance of Sorafenib in HCC Cells and Enhances the Sensitivity of HCC Cells to Sorafenib by Targeting cyp3a4
Source: Front Oncol. 2021 Jul 26;11:735447. doi: 10.3389/fonc.2021.735447 (PMC8350395; doi:10.3389/fonc.2021.735447)
Supplement: Supplementary file 1 [file Table_1.doc]

Supplemental Table 1 Using HPLC to confirm the purity of the drugs involved in this research

| Compounds | purity (%) |
| --- | --- |
| sorafenib | 99.1 |
| cabozantinib | 99.5 |
| regorafenib | 99.3 |
| lenvatinib | 99.2 |
| ketoconazole | 99.1 |
| amprenavir | 99.4 |
| diltiazem | 99.2 |
